# Measuring the Effects of Climate Change on Wheat Production: Evidence from Northern China

**DOI:** 10.3390/ijerph191912341

**Published:** 2022-09-28

**Authors:** Huaquan Zhang, Yashuang Tang, Abbas Ali Chandio, Ghulam Raza Sargani, Martinson Ankrah Twumasi

**Affiliations:** College of Economics, Sichuan Agricultural University, Chengdu 611130, China

**Keywords:** wheat production, climate change, northern region, China

## Abstract

The current study examines the long-run effects of climatic factors on wheat production in China’s top three wheat-producing provinces (Hebei, Henan, and Shandong). The data set consists of observations from 1992 to 2020 on which several techniques, namely, fully modified OLS (FMOLS), dynamic OLS (DOLS), and canonical co-integrating regression (CCR) estimators, and Granger causality, are applied. The results reveal that climatic factors, such as temperature and rainfall, negatively influenced wheat production in Henan Province. This means that Henan Province is more vulnerable to climate change. In contrast, it is observed that climatic conditions (via temperature and rainfall) positively contributed to wheat production in Hebei Province. Moreover, temperature negatively influenced wheat production in Shandong Province, while rainfall contributed positively to wheat production. Further, the results of Granger causality reveal that climatic factors and other determinants significantly influenced wheat production in the selected provinces.

## 1. Introduction

China cultivates only eight percent of the world’s arable land to feed eighteen percent of the global population. It is expected that China’s population will peak around 2030 [1]; hence, the food security problem in China has always been a concern. In the “Outline of the 14th Five-Year Plan for National Economic and Social Development of the People’s Republic of China and the Long-term Goals in 2035,” the Chinese government, for the first time, incorporated the food security strategy into the planning system and set the goal of ensuring that grain output will remain stable at over 650 million tons over the 14th Five-Year Plan period. Climate has a strong influence on food production. Climate change and more frequent bad weather events around the world exacerbate the insecurity of China’s grain production [2,3]. As a result, understanding the impact of climate change on China’s grain crop productivity and devising countermeasures to implement China’s food security strategy are critical.

The impact of climate change on grain crop production has both advantages and disadvantages. Nevertheless, the disadvantages outweigh the advantages overall, and different climate variables have different impacts on different crops and regions [4,5,6]. In recent years, the increased heat caused by climate change has been conducive to expanding the grain sown area and producing more grain [7]. Increasing rainfall and CO_2_ concentrations are beneficial for crop production to some extent, but high temperatures may negate this effect in some areas [8,9]. Similarly, climate change had a negative impact on grain production by expanding pest and disease occurrence areas, shortening crop growth cycles, and increasing the frequency of extreme weather events [10,11].

The global climatic variations are a sensitive topic being discussed in China. According to the National Meteorological Administration, China’s temperature has increased by 0.3 °C every 10 years (higher than the global average during the same period), and its annual precipitation has increased by 5.1 mm every 10 years between 1961 and 2020 [12]. Climate change is causing a “double increase in water and heat.” By evaluating the expected impact of global warming on the yields of China’s main crops (wheat, rice, and corn), it was discovered that the crop yield effect emanating from climate change is primarily due to an increase in air temperature [13]. Grain production has been impacted by significant deviations in China’s agricultural climate resources. The regional space of grain production is also changing, with the emphasis shifting to the main production areas in the north [14]. Researchers incorporate technological progress factors into the research system of food production under climate change [15,16,17,18]. Food security is a technical issue in terms of production mode, and the level of technology determines the ability to ensure food security. As a result, technological advancement can be used as an effective tool for grain production to cope with climate change [19,20].

Many studies have found that technological progress significantly impacts grain production, which is primarily reflected in the two factors listed below. First, technological advancement has improved the crops’ ability to withstand disasters. The advancement of bio-pesticides due to technological advancement has increased the agricultural departments’ ability to prevent and control major agricultural pests and diseases while causing less environmental impact [21,22]. Simultaneously, the monitoring technology system of major sudden agricultural meteorological disasters constructed by the agricultural sector is also conducive to the agricultural sector’s better response to extreme weather events [23]. Second, technological advancement is a driving force in changing the mode of production. Modern agricultural mechanization can promote rapid agricultural production growth while producing good environmental results [24,25]. Fertilizer use can improve crop yield, but excessive use reduces crop yield [26]. Improved seed varieties, fertilizers, pesticides, technical equipment, and infrastructure are used as proxies for technological progress in agricultural production [18,27,28,29]. Hence, this study also considers fertilizer use a proxy for technological advancement, as it is a crucial factor in crop production.

**Figure 1:**
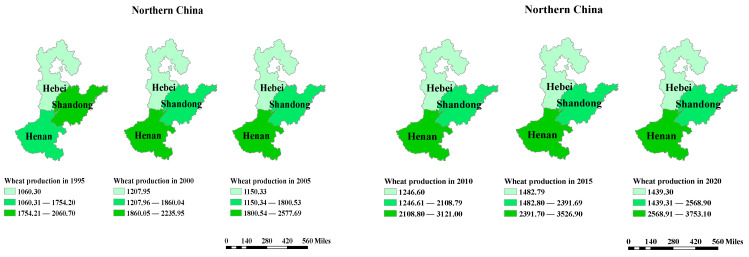
The distribution of wheat production (10,000 tons) in Hebei, Henan, and Shandong Provinces.

**Figure 2:**
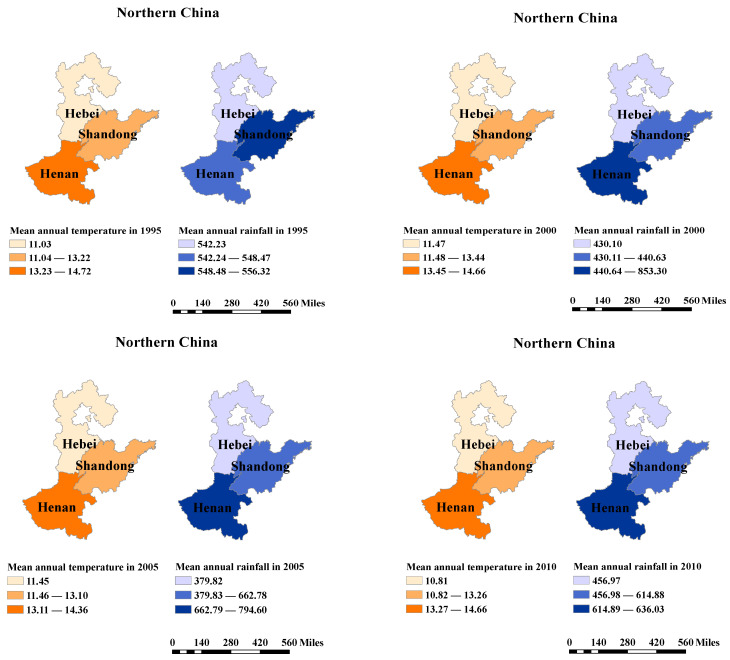

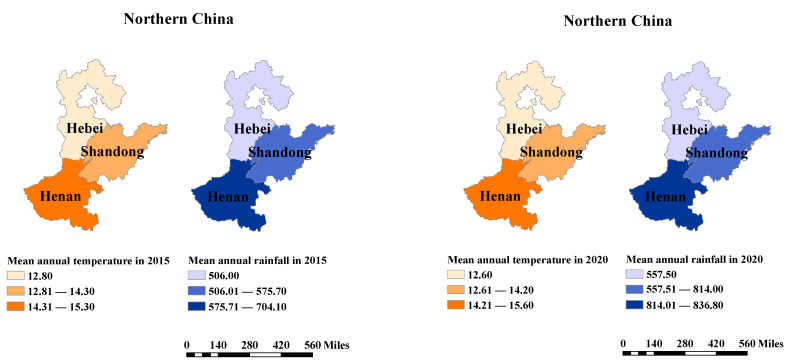
The distribution of temperature (°C) and rainfall (mm) in Hebei, Henan, and Shandong Provinces.

Wheat is China’s second most important food crop, after rice, and its planting area accounts for 22~30% of the total cultivated land [30]. Winter wheat is the predominant crop in China, accounting for more than 90 percent of the total output [31]. The regions of China that produce winter wheat can be divided into *Southern* and *Northern* cultivation areas. The northern winter wheat producing region is the most concentrated wheat growing and consuming region in China, and its sown area and wheat output account for around two-thirds of the country. In recent years, the top three provinces in northern China for winter wheat production were Henan, Shandong, and Hebei. Henan Province has an alternating temperate monsoon and subtropical climate, whereas Shandong and Hebei Provinces have a temperate monsoon climate. Figure 1 and Figure 2 show their geographical location, wheat yield, temperature, and rainfall.

**Figure 3:**
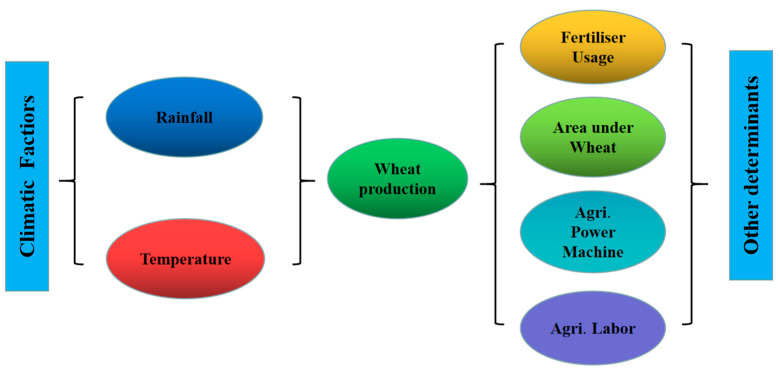
The study’s conceptual framework.

Previous studies in various parts of the world have extensively documented the impact of changing climate on wheat crop yield. Most existing research on China discussed the relationship between the two at the national and regional levels [31,32,33] or only focused on a specific province, such as Henan Province, which has the highest wheat yield [30,34]. However, climate change causes food production variability in regions with varying climate resources. The current paper assesses the long-term effects of changing climate on wheat production in the top three provinces in northern China to further analyze the heterogeneous influence of changing climate on wheat production, propose targeted measures to deal with climate change for the main grain-producing areas in China, and contribute to China’s food security strategy. Figure 3 shows the dynamic nexus between climatic factors, other determinants, and wheat production.

## 2. Literature Review

Wheat may be one of the crops most susceptible to the effects of climate change, but its substantial impact on crop production is profound. Numerous research works have investigated the impacts of climate change on wheat development and harvest in major wheat-producing regions in Asia, Europe, and northern Africa. However, it is important to note that varied temperature conditions and precipitation patterns affect wheat growth and yield differently in different regions.

Several researchers, for example, Zhai et al. [18], Abbas [27], Gul et al. [28], Ali et al. [35], and Warsame et al. [36], have explored the short-term and long-term climate change effects on food crop production by applying the autoregressive distributed lag (ARDL) methodology and reported mix outcomes related to climate variables. While, a study by You et al. [37] revealed that climate warming lowered wheat yield growth, a 1 °C rise in wheat growing temperature reduced wheat production by 3–10% in China. However, the findings of Zhai et al. [18] from 1970 to 2014 in China evaluated that temperature did not significantly influence the amount of wheat produced per unit of land in the short run and long run, while farm mechanization and fertilizer usage increased wheat output in the long run.

More recently, the research by Gul et al. [5] from 1985 to 2016 in Pakistan established the long-term link between climate variables and main food crop production. The findings showed that temperature negatively affects key food crop production, while rainfall improves food production. Similarly, the study of Chandio et al. [38] from 1977 to 2014 in Pakistan revealed that climate change and CO_2_ have a detrimental short- and long-term influence on grain productivity, reducing cereal production and causing food security issues in the country.

Another similar study by Warsame et al. [39] for the period of 1980–2017 in Somalia examined the effects of climate change along with political instability on the productivity of sorghum by using various estimation techniques (i.e., FMOLS, DOLS, and CCR). The findings revealed that political instability and temperature significantly declined the productivity of sorghum, while rainfall and cultivated area enhanced the production in the long term. The long-term findings are also verified by the CCR approach. In the case of India, Bhardwaj et al. [40] reported that climate variables negatively contributed to wheat and paddy production; moreover, excessive rainfall had a detrimental influence on wheat and rice yields.

In the case of Ghana, Ntiamoah et al. [41] used a novel dynamic simulated autoregressive distributed lag (ARDL) model to examine the impact of CO_2_ emissions, rainfall, credit supply, and fertilizer on the productivity of maize and soybean, covering the period from 1990 to 2020. The findings revealed that CO_2_ emissions, as well as rainfall, have a significant and positive impact on crop production, while the supply of credit and fertilizer negatively influenced maize production. In the context of Asia, Ozdemir [42] studied the effects of climate change on agricultural output by using various estimation techniques (i.e., PMG and CCEMG). The outcomes showed that temperature and CO_2_ affected agricultural output negatively and significantly in the long term, while precipitation improved productivity. In addition, other factors, such as power consumption for agricultural machinery and fertilizer, significantly enhanced agricultural output in the same period.

According to research by Akhtar and Masud [4] on the influence of climatic factors on rice and cereal production from the period 1985–2016, it was found that temperature and energy usage severely influence rice and vegetable output, although their effect on cereal productivity is minor. However, CO_2_ emissions negatively influenced coffee production, and the temperature, energy use, and fossil fuel usage induced climate change, which had a negative impact on Malaysian agriculture. The empirical study of Kumar et al. [43] in selected lower-middle-income nations from 1971 to 2016 assessed the climate change–cereal crops production association. The authors’ findings revealed that rising temperatures diminish crop productivity. Rainfall and CO_2_ emissions boosted crop yields, and a bidirectional causation between grain output, temperature, and CO_2_ emissions was discovered. Rainfall and temperature affect grain production uni-directionally and might threaten the food security of the rural populations.

In particular, a study in China by Pickson et al. [44] from 1998 to 2017 examined the effects of climate change on rice cultivation. The findings showed that the climate variable, such as the temperature, adversely influences rice cultivation, while average rainfall influences the rice output but is insignificant. The farmed area positively affected short-term crop output. At the same time, fertilizer use had little effect and bidirectional causation between rice production and the cultivated area. Similarly, the investigation of Pickson et al. [6] in China over the period 1990Q1–2013Q4 found that the average temperature and its variability associated with cereal production were negative but significant in the long run. Additionally, rainfall variability and cereal production linkage showed no significant effect in the long run, but two variables (CO_2_ and temperature variability) had a negatively significant association in the short run.

Likewise, Pickson et al. [45] investigated the impact of global warming on the main food crops (i.e., rice and maize) production in the case of China over the periods 1978Q1–2015Q4. The outcomes revealed a significantly positive trend in average temperature and seasonal temperature increases during the spring, summer, and fall with the insignificant change in the monthly, seasonal, and annual precipitation. The impact of temperature decreases maize and rice production at higher quantiles.

Due to the diverse time scales, geographic locations, and techniques, the following research work has not yet formed consistent findings on climate change’s influence on wheat growth and yield. These research works failed to combine the climatic conditions and agricultural progress elements into crop yield–climate functions to investigate their influence, and the long-run impacts on wheat must be studied. In this work, we employed the FMOLS method to assess the long-run climate variations’ influence on wheat yield in the northern region of China. The findings of the FMOLS approach are verified by the DOLS and CCR estimators.

## 3. Materials and Methods

### 3.1. Data

The present study intends to investigate the long-run impact of temperature, rainfall, fertilizer usage, power usage, farming area, and labor on wheat production in the context of top 3 wheat-producing provinces of northern China. Wheat production is used as the dependent variable, and it is measured in 10,000 tons, while climatic factors, namely, temperature measured in degrees Celsius, rainfall measured in millimeters, fertilizer usage measured in 10,000 tons, power consumption measured in 1000 kWh, the cultivated area measured in 1000 hectares, and labor measured in 10,000 people, are used as the independent variables. The data were extracted from the China Rural Statistical Yearbook (https://data.cnki.net/Yearbook/Navi?type=type&code=A# (accessed on 1 June 2022)) and the National Weather Science Data Center (http://data.cma.cn/ (accessed on 1 June 2022)).

### 3.2. Econometric Modeling

This study examines the long-run impact and the causal relationship between temperature, rainfall, fertilizer usage, power consumption, cultivated area, labor, and wheat production in the selected provinces of China. Several investigation tests were carried out to achieve the research objective, including the co-integration test, FMOLS, DOLS, and CCR estimators, and the Granger causality test. The basic model is constructed as shown below:

(1) $$WP=f\left(TEMP,\;RF,\;FER,\;PC,\;WA,LF\right)$$

The logarithmic arrangement of Equation (1) can be developed as follows:

(2) $$LWP_{t}=\varphi_{0}+\varphi_{1}LTEMP_{t}+\varphi_{2}LRF_{t}+\varphi_{3}LFER_{t}+\varphi_{4}LPC_{t}+\varphi_{5}LWA_{t}+\varphi_{6}LLF_{t}+\varepsilon_{t}$$

where *WP* indicates wheat production, *TEMP* denotes average annual temperature, *RF* represents average annual rainfall, *FER* indicates fertilizer usage, *PC* shows the power consumption, *WA* stands for wheat cultivated area, *LF* indicates rural labor force.

### 3.3. FMOLS Long-Run Estimator

This paper uses the fully modified OLS (FMOLS) proposed by Phillips and Hansen [46] to estimate the co-integration coefficient. Based on the OLS, this method uses the semi-parametric two-stage estimation method to correct the equalization error and the explained variable, which can effectively eliminate the endogeneity caused by the co-integration relationship and the sequence correlation of error terms, thus obtaining the consistent estimator of co-integration parameter estimator and the asymptotic normal distribution of FMOLS estimator. Suppose the model is

(3) $$Y_{t}={\theta X}_{t}+\mu_{1t}$$

(4) $${\Delta X}_{t}=\mu_{2t}$$

Let $\mu_{t}^{\prime }=\mu_{1t}^{\prime }+\mu_{2t}^{\prime }$. First, perform OLS estimation on Equation (3) to obtain the $\hat{\theta }$ and $\hat{\mu_{t}}$ of OLS estimators. Second, estimate the long-term variance of $\mu_{t}$. Let Ω and ∆ represent long-term variance and one-sided long-term variance, respectively, and the estimates are shown in Formulae (5) and (6).

(5) $$\Omega =\overset{\infty }{\underset{i=-\infty }{\sum }}E\left(\mu_{t}\mu_{t-1}^{\prime }\right)=\left[\begin{array}{cc} a_{11} & a_{12}\\ a_{21} & a_{22} \end{array}\right]$$

(6) $$\Delta =\overset{\infty }{\underset{i=0}{\sum }}E\left(\mu_{t}\mu_{t-1}^{\prime }\right)=\left[\begin{array}{cc} b_{11} & b_{12}\\ b_{21} & b_{22} \end{array}\right]$$

By adjusting the endogeneity, we can obtain Equation (7).

(7) $$\hat{Y_{t}^{+}}=Y_{t}-\hat{a_{12}}\;{\hat{a_{22}}}^{-1}{\Delta X}_{t}$$

By adjusting the sequence correlation, we can obtain Equation (8).

(8) $$\hat{b_{12}^{+}}=\hat{b_{12}}-\hat{a_{11}}\;{\hat{a_{22}}}^{-1}\;\hat{b_{22}^{\prime }}$$

The final FMOLS estimator is obtained as shown in Equation (9).

(9) $$\hat{\theta^{+}}=\left[\overset{T}{\underset{t=1}{\sum }}\hat{Y_{t}}+X_{t}^{\prime }-T\left(\hat{b_{12}^{+}\;},\;0\right)\right]{\left(\overset{T}{\underset{t=1}{\sum }}X_{t}X_{t}^{\prime }\right)}^{-1}$$

Furthermore, we use the dynamic OLS (DOLS) proposed by Stock and Watson [47] and the canonical co-integrating regressions (CCR) proposed by Park [48] to verify the robustness of FMOLS estimation results. The DOLS estimation model contains the lag term of explanatory variables, and the standard deviation of its estimator has a normal asymptotic distribution, which is also better than the OLS estimation [40]. The idea of the CCR model is the same as the FMOLS model, but the difference is that it transforms the data stationarity to obtain the least-square estimation and then eliminates the dependency between the co-integration equation and the random correction equation of the explanatory variables.

## 4. Results and Discussion

**Figure 4:**
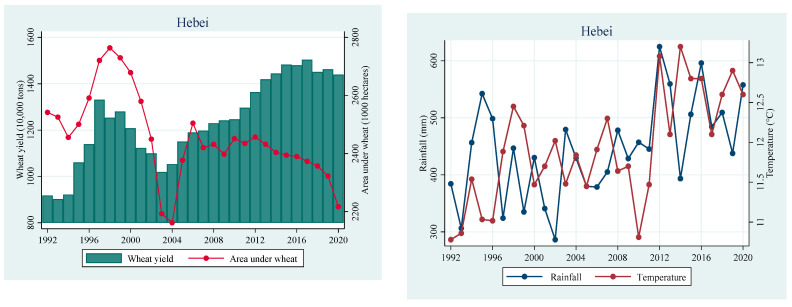

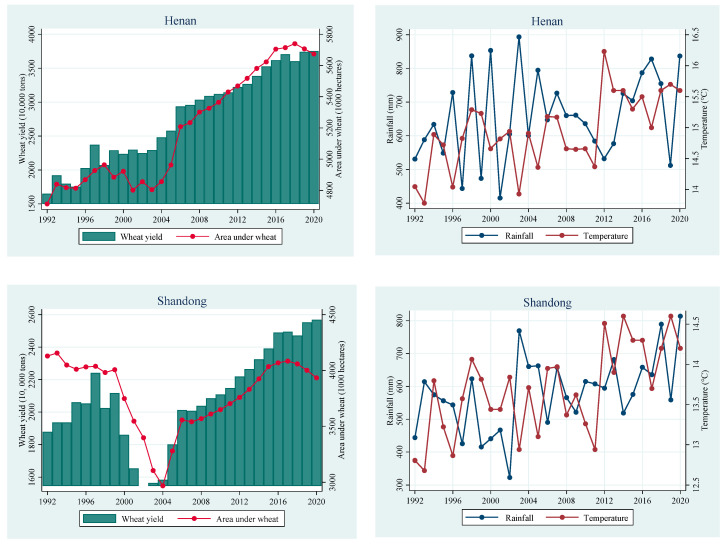
Trend of wheat production, temperature, and rainfall in Hebei, Henan, and Shandong Provinces of China.

We use the FMOLS, DOLS, and CCR estimators to examine the long-term influence of temperature, rainfall, fertilizer use, power consumption, wheat farming area, and labor on wheat production in the selected three provinces of China. Table 1 reports the statistical summary of the dependent and independent variables for the Hebei, Henan, and Shandong Provinces. The J-B test confirms that all the studied variables are normally distributed. Figure 4 shows the trend of wheat production and climatic factors in the selected provinces of China.

The outcomes of the correlation matrix for Hebei, Henan, and Shandong Provinces are presented in Table 2. The findings for Hebei Province reveal that temperature, rainfall, fertilizer usage, power consumption, and labor are significantly and positively associated with wheat production, while the cultivated area is negatively associated. Further, the findings for Henan Province show that all the studied variables are significantly linked with wheat production, except rainfall. In addition, the outcomes for Shandong Province indicate that temperature, cultivated area, and labor are significant and interrelated with wheat production, whereas fertilizer is negatively associated.

We employ the Johansen and Juselius co-integration procedure to explore the long-term association between the explained variables, such as wheat production and its explanatory variables. We test the research hypothesis, as the null hypothesis states that the explained variables, wheat production and its explanatory variables, are not co-integrated in the long term. In contrast, the alternative hypothesis mentions that the considered variables are co-integrated in the long term. To reach a decision about the hypothesis, we use the Trace t-statistic test, and the findings for Hebei, Henan, and Shandong Provinces are reported in Table 3. The findings reveal that wheat production, temperature, rainfall, fertilizer usage, power consumption, cultivated area, and labor force are co-integrated in the long term in China’s selected wheat-producing provinces.

Table 4 reports the estimated results of long-term effect of climate variables and other control variables on wheat yield in Hebei, Henan, and Shandong Provinces, respectively.

In the case of Hebei Province, the climate variables (i.e., temperature and rainfall) have a positive, significant impact on wheat production. This means the climate conditions are more favorable for wheat cultivation in Hebei Province. Specific to the North China Plain, where this study area is located, some research evidence shows that in the north of this plain, the impact of rainfall on wheat production is positive, while in the south of this plain, the impact of rainfall turns negative [49]. Similarly, for temperature, the increase in temperature increases the winter wheat yield in the northern part of the North China Plain but decreases the wheat yield produced in winter in the south of the North China Plain [50]. The top three wheat-producing provinces are selected for this investigation. Hebei, Shandong, and Henan Provinces are distributed in the North China Plain. The three provinces’ yearly mean temperature and yearly mean precipitation are ranked from low to high in Hebei, Shandong, and Henan (see Figure 2). The temperature and rainfall in Hebei Province are low, and the impacts of temperature and precipitation on wheat yield are positive.

Further results reveal that fertilizer use, cultivated area, and labor force also have a positive, significant influence on wheat production. The long-run coefficients of fertilizer use, cultivated area, and labor force indicate that a 1% increase in fertilizer treatment use, cultivated area, and labor force wheat production improved by 0.17%, 0.58%, and 1.36%, respectively.

In the case of Henan Province, the climatic factors (i.e., temperature and rainfall) and wheat production relationship was significant and negative. This means that climatic factors severely impact wheat production in Henan Province. The long-run coefficient of both climate variables, temperature and rainfall, indicates that with a 1% increase in both climate variables (i.e., temperature and rainfall), wheat production decreases by 0.51%, and 0.05%. Geng et al. [51] reported that high temperatures will be detrimental to wheat production by shortening the growth cycle of the wheat crop. Further, Song et al. [33] stated that excessive rainfall causes excessive water accumulation, which will aggravate the wet damage of wheat and negatively affect wheat production.

Moreover, the results show that fertilizer usage also significantly negatively impacts wheat production. The long-term coefficient of fertilizer usage reveals that if a farmer overuses the fertilizer by 1%, wheat production declines by 0.61%. Fertilization can not only supplement the nutrients needed by wheat but also improve the utilization rate of water, thus increasing the yield of wheat [52,53]. However, unreasonable and excessive use of chemical fertilizers will cause soil degradation and adversely affect wheat yield. This shows that the rational use of chemical fertilizers is very important for wheat production, and Henan Province should pay more attention to improving chemical fertilizer use efficiency.

In contrast, these variables (power usage, wheat farming area, and labor force) and the wheat production relationship were significant and positive. The long-run coefficient of power usage, wheat farming area, and labor force reveals that a 1% increase in power usage, wheat farming area, and labor force increases wheat production by 0.48%, 2.98%, and 0.21%, respectively. 

In the case of Shandong Province, the climate variables, temperature, and wheat production displayed a diverse relationship. At the same time, rainfall had a significant and positive influence, suggesting that with a 1% increase in temperature and rainfall, wheat production decreased by 0.07% and improved by 0.08%. The heterogeneous effect of the climate variables on regional wheat yield is verified by some existing studies. For example, the evidence from Mexico and China verified that the sensitivity of wheat yield to climate variables is uneven in space [54,55]. Tao et al. [56] studied climate change’s influence on wheat productivity and found the prospective consequences of climate change on winter wheat output in northern China under 10 climatic scenarios and concluded that environmental variability might enhance wheat yield by 37.7% (18.6%), 67.8% (23.1%), and 87.2% (34.4%), with (without) CO_2_ fertilization effects in the 2020s, 2050s, and 2080s, respectively, in the future. The temperature and rainfall in Shandong Province are in the middle of the three provinces, and the impact of temperature on wheat yield is negative, but the impact of rainfall is positive. In Henan Province, it is observed that the temperature is higher, and the rainfall is higher; the influence of temperature and rainfall on wheat is negative. Moreover, the results show that these variables (fertilizer use, cultivated area, and labor force) and wheat production association was significant and positive, suggesting that a 1% increase in fertilizer usage, cultivated area, and labor force enhanced wheat production by 0.29%, 1.16%, and 0.23%, respectively.

This study applied the DOLS and CCR long-run estimators as a robust check approach for the FMOLS findings. Table 5 shows that climate variables positively affect wheat production in the context of Hebei Province. The estimated coefficients of DOLS and CCR are consistent with the findings of the FMOLS model. Likewise, in Henan Province’s case, climatic factors negatively influence wheat production. These outcomes are also consistent with the outcomes of the FMOLS model. In addition, climatic factors, such as temperature, only have a negative impact on wheat production. Meanwhile, rainfall has a significant and positive linkage with wheat production. Hence, the results of both techniques, such as DOLS and CCR, are similar to the results of the FMOLS method.

Although the long-run impact of the variables concerned was explored through the FMOLS, DOLS, and CCR estimators, the causal connection between the underlying variables is still in question. Therefore, we further apply the Granger causality method. The findings for Hebei, Henan, and Shandong Provinces are presented in Table 6. A bidirectional causality between precipitation and fertilizer usage with wheat production in the context of Hebei Province can be observed. This means that rainfall and fertilizer usage significantly contributed to Hebei Province’s wheat production.

Further, the results only discover a unidirectional causality association between wheat production and temperature. In the context of Henan Province, it is revealed that a unidirectional causality association runs from precipitation and fertilizer usage to wheat production. In contrast, a bidirectional causality exists between power consumption and wheat production. This depicts that climate change factors, such as rainfall, and other inputs also positively influence wheat production. In addition, a bidirectional causality is established between the farming area and wheat production, while a unidirectional causality is detected from precipitation and labor to wheat production. These results imply that the cultivated area, rainfall, and labor significantly improve wheat production in the context of Shandong Province.

## 5. Conclusions

The current study assesses the climate variables’ long-run impact on wheat production in China’s top three wheat-producing provinces. The other important factors considered in this paper include fertilizer usage, cultivated area, power consumption, and labor. The data set consists of observations from 1992 to 2020 on which several time-series techniques, namely, the DOLS, FMOLS, CCR, and Granger causality, were applied. Based on the estimations, the findings revealed that wheat production is negatively affected by climate change in Henan Province. In contrast, climate change is more favorable for wheat production in Hebei Province.

On the other hand, temperature negatively influenced wheat production but was not significant, while rainfall significantly contributed positively to wheat production in Shandong Province. Further findings showed that fertilizer usage, cultivated area, and labor positively and significantly improved wheat production in Hebei and Shandong Provinces. In contrast, power usage, wheat farming area, and labor force significantly and positively enhanced wheat production in Henan Province. In addition, the findings of the Granger causality test reported a bidirectional causality between rainfall and fertilizer use with wheat production in Hebei Province, while a unidirectional causality connection was revealed between wheat production and temperature. In the context of Henan Province, it was discovered that a unidirectional causality link was observed from rainfall and fertilizer use to wheat production. In contrast, a bidirectional causality existed between power consumption and wheat production. Moreover, a bidirectional causality was established between the cultivated area and wheat production, while a unidirectional causality was detected from the rainfall and labor to wheat production in Shandong Province.

Based on the estimated outcomes, the current paper offers several policy implications:

With both advantages and disadvantages, China’s wheat production is affected by global warming. To mitigate the effects of a changing climate on China’s wheat yield, it is vital to increase the adaptability of wheat production. First, modify wheat’s sowing date and area in a reasonable manner. Adjust the sowing date of crops, rationally plan the planting areas, fully utilize the additional heat resources brought about by climate change, decrease the impact of meteorological disasters, and increase the stability of wheat production based on the climatic conditions of various regions.

Second, agricultural technology advancement will continue to be important in ensuring wheat yield stability. On the one hand, the Chinese government must prioritize research and develop seed resources resistant to extreme weather conditions. It is crucial to develop and store wheat germplasm resources that can respond to adverse weather conditions, given the prevalence of extreme weather events (high-temperature resistance, waterlogging resistance, low-temperature resistance, etc.). On the other hand, it is essential to continue using advanced agricultural technologies to produce wheat. For instance, more fertilizer use techniques should be implemented to increase the input effectiveness of chemical fertilizers and ensure the sustainability of agricultural production.

Furthermore, there are regional differences in wheat planting varieties and methods in China, making it difficult to continuously improve wheat production levels by relying solely on a single technology. As a result, it is necessary to promote improved varieties in conjunction with good methods, agricultural machinery, and agronomy, as well as to further tap the potential of science and technology to increase production.

## Figures and Tables

**Table 1:** Statistical summary for Hebei, Henan, and Shandong Provinces.

Hebei Province							
	LWP	LTEMP	LRF	LFER	LPC	LWA	LLF
Mean	3.1027	1.0793	2.6475	2.4727	3.9040	3.3885	3.4696
Median	3.0969	1.0779	2.6492	2.4830	3.8975	3.3859	3.4767
Maximum	3.1772	1.1205	2.7955	2.5258	4.0454	3.4415	3.5288
Minimum	3.0081	1.0338	2.4567	2.3438	3.6371	3.3347	3.4105
Std. Dev.	0.0515	0.0235	0.0839	0.0444	0.1032	0.0272	0.0387
Skewness	−0.1277	−0.0406	−0.3541	−0.9106	−0.6792	0.1607	−0.1323
Kurtosis	1.8645	2.1997	2.6445	3.6768	3.1674	2.8598	1.5297
J-B	1.4673	0.7008	0.6804	4.0902	2.0299	0.1332	2.4176
Prob.	0.4801	0.7043	0.7115	0.1293	0.3624	0.9355	0.2985
Obs.	26	26	26	26	26	26	26
**Henan Province**							
	**LWP**	**LTEMP**	**LRF**	**LFER**	**LPC**	**LWA**	**LLF**
Mean	3.4487	1.1764	2.8157	2.7334	3.8913	3.7184	3.6723
Median	3.4766	1.1752	2.8199	2.7675	3.9574	3.7216	3.6727
Maximum	3.5743	1.2103	2.9511	2.8549	4.0685	3.7589	3.7319
Minimum	3.2440	1.1439	2.6183	2.5081	3.4935	3.6813	3.5766
Std. Dev.	0.0966	0.0158	0.0917	0.1129	0.1639	0.0288	0.0441
Skewness	−0.3490	−0.0922	−0.4660	−0.5676	−0.9815	0.0464	−0.5964
Kurtosis	1.9237	2.6353	2.3330	1.9491	2.8955	1.4106	2.5309
J-B	1.7827	0.1809	1.4228	2.5924	4.1870	2.7458	1.7800
Prob.	0.4100	0.9135	0.4909	0.2735	0.1232	0.2533	0.4106
Obs.	26	26	26	26	26	26	26
**Shandong Province**							
	**LWP**	**LTEMP**	**LRF**	**LFER**	**LPC**	**LWA**	**LLF**
Mean	3.3183	1.1379	2.7570	2.6420	3.9461	3.5722	3.5979
Median	3.3215	1.1386	2.7671	2.6487	3.9937	3.5792	3.6005
Maximum	3.4097	1.1643	2.9106	2.6992	4.1255	3.6110	3.6511
Minimum	3.1895	1.1093	2.5093	2.5590	3.6038	3.4724	3.5520
Std. Dev.	0.0652	0.0163	0.0922	0.0389	0.1504	0.0378	0.0352
Skewness	−0.5563	−0.0943	−0.6423	−0.5870	−0.9727	−1.0561	0.1103
Kurtosis	2.4971	2.0359	3.4293	2.2740	2.8985	3.4102	1.4679
J-B	1.6152	1.0454	1.9874	2.0641	4.1115	5.0157	2.5956
Prob.	0.4459	0.5929	0.3701	0.3562	0.1279	0.0814	0.2731
Obs.	26	26	26	26	26	26	26

Note: LWP, LTEMP, LRF, LFER, LPC, LWA, LLF signify the natural log of wheat production, average annual temperature, average annual rainfall, fertilizer usage, power consumption, wheat cultivated area, and rural labor force, while J-B denotes the Jarque–Bera test.

**Table 2:** Results of the correlation matrix for Hebei, Henan, and Shandong Provinces.

Hebei Province							
	LWP	LTEMP	LRF	LFER	LPC	LWA	LLF
LWP	1.0000						
LTEMP	0.6897 ***	1.0000					
LRF	0.3649 *	0.1626	1.0000				
LFER	0.5910 ***	0.4553 **	0.2603	1.0000			
LPC	0.3733 *	0.3439 *	0.1293	0.9014	1.0000		
LWA	−0.0362	−0.1602	−0.3598 *	−0.3973 **	−0.4116 **	1.0000	
LLF	0.6651 **	0.3895 **	0.4921 **	0.8690 ***	0.7928 ***	−0.4726 **	1.0000
**Henan Province**							
	**LWP**	**LTEMP**	**LRF**	**LFER**	**LPC**	**LWA**	**LLF**
LWP	1.0000						
LTEMP	0.5545 ***	1.0000					
LRF	0.1796	−0.1576	1.0000				
LFER	0.9509 ***	0.4881 **	0.2164	1.0000			
LPC	0.9201 ***	0.4610 **	0.1996	0.9793 ***	1.0000		
LWA	0.9520 ***	0.6116 ***	0.2225	0.8954 ***	0.8180 ***	1.0000	
LLF	0.8656 ***	0.5235 ***	0.1868	0.8916 ***	0.8873 ***	0.7996 ***	1.0000
**Shandong Province**							
	**LWP**	**LTEMP**	**LRF**	**LFER**	**LPC**	**LWA**	**LLF**
LWP	1.0000						
LTEMP	0.5323 ***	1.0000					
LRF	0.3144	0.0439	1.0000				
LFER	−0.1036	0.1261	0.0995	1.0000			
LPC	0.2986	0.4270 **	0.3541 *	0.7304 ***	1.0000		
LWA	0.8281 ***	0.3793 **	−0.048	−0.4079 **	−0.144	1.0000	
LLF	0.7342 ***	0.5986 ***	0.4947 **	0.3109	0.8066 ***	0.3404 *	1.0000

Note: *** *p* value < 0.01, ** *p* value < 0.05, and * *p* value < 0.1.

**Table 3:** Co-integration outcomes for Hebei, Henan, and Shandong Provinces.

Hebei Province		Henan Province		Shandong Province	
Rank	TS	Rank	TS	Rank	TS
None *	242.8063 (0.0000)	None *	250.6836 (0.0000)	None *	245.8051 (0.0000)
At most 1 *	151.8709 (0.0000)	At most 1 *	143.9006 (0.0000)	At most 1 *	165.5126 (0.0000)
At most 2 *	91.0448 (0.0004)	At most 2 *	98.4322 (0.0001)	At most 2 *	95.6523 (0.0001)
At most 3 *	54.7745 (0.0098)	At most 3 *	59.4932 (0.0028)	At most 3 *	60.6158 (0.0020)
At most 4	24.8771 (0.1659)	At most 4 *	34.6498 (0.0128)	At most 4	29.5732 (0.0531)
At most 5	6.4268 (0.6451)	At most 5	13.2864 (0.1047)	At most 5	14.3444 (0.0740)
At most 6	0.0618 (0.8036)	At most 6	3.6392 (0.0564)	At most 6	3.8247 (0.0505)

Note: TS indicates the trace statistic, * signifies rejection of the hypothesis at the 0.05 level.

**Table 4:** Results of FMOLS estimator for top three provinces in northern China.

Variables	Hebei Province		Henan Province		Shandong Province	
	Coefficient	Prob.	Coefficient	Prob.	Coefficient	Prob.
LTEMP	1.1600 ***	0.0000	−0.5129 *	0.0929	−0.0701	0.7446
LRF	0.0136	0.8277	−0.0576	0.1387	0.0823 *	0.0522
LFER	0.1726	0.5623	−0.6117 **	0.0325	0.2917 *	0.0695
LPC	−0.3626 ***	0.0009	0.4885 ***	0.0037	−0.1421 *	0.0980
LWA	0.5805 ***	0.0019	2.9805 ***	0.0000	1.1690 ***	0.0000
LLF	1.3669 ***	0.0000	0.2161	0.1962	0.2351	0.7447
C	−3.9019 ***	0.0001	−7.8904 ***	0.0000	−2.1196	0.3335
R^2^	0.8061		0.9722		0.9332	
Adj-R^2^	0.7415		0.9630		0.9058	

Note: *** *p* value < 0.01, ** *p* value < 0.05, and * *p* value < 0.1.

**Table 5:** Robustness check.

	Hebei Province		Henan Province		Shandong Province	
	DOLS	CCR	DOLS	CCR	DOLS	CCR
Variables	Coefficient	Coefficient	Coefficient	Coefficient	Coefficient	Coefficient
LTEMP	1.3956 *** (0.0003)	1.3629 *** (0.0000)	−0.2405 (0.4173)	−0.6182 (0.1652)	−0.1411 (0.7269)	−0.0961 (0.4601)
LRF	0.0837 (0.4661)	0.0293 (0.6728)	−0.0107 (0.8369)	−0.0187 (0.7913)	0.4315 * (0.0791)	0.1276 *** (0.0054)
LFER	0.2372 (0.5224)	0.1416 (0.5718)	−0.5957 * (0.0900)	−0.7901 ** (0.0292)	0.1267 (0.7971)	0.3789 *** (0.0033)
LPC	−0.2898 ** (0.0213)	−0.3205 *** (0.0002)	0.1385 (0.4038)	0.5703 *** (0.0023)	0.0943 (0.7438)	−0.1125 * (0.0580)
LWA	0.5525 *** (0.0038)	0.5663 *** (0.0000)	2.2683 ** (0.0167)	3.1978 *** (0.0000)	1.6290 ** (0.0153)	1.4074 *** (0.0000)
LLF	1.0557 *** (0.0081)	1.2416 *** (0.0000)	−0.2059 (0.4506)	0.2741 (0.1504)	−0.4386 (0.7924)	−0.8140 * (0.0798)
C	−3.6129 *** (0.0016)	−3.7710 *** (0.0000)	−2.9953 (0.3278)	−8.7276 *** (0.0000)	−2.6588 (0.5218)	0.3107 (0.8046)
R^2^	0.9402	0.7980	0.9922	0.9662	0.9881	0.9251
Adj-R^2^	0.8805	0.7307	0.9814	0.9549	0.9454	0.8943

Note: *** *p* value < 0.01, ** *p* value < 0.05, and * *p* value < 0.1.

**Table 6:** Granger causality test outcomes for Hebei, Henan, and Shandong Provinces.

Null Hypothesis:	Hebei Province		Henan Province		Shandong Province	
	F-Statistic	Prob.	F-Statistic	Prob.	F-Statistic	Prob.
LTEMP ⇏ LWP	0.32014	0.7299	0.48642	0.4928	6.9 × 10^−5^	0.9934
LOGWP ⇏ LTEMP	3.85467 **	0.0393	7.70193 **	0.0110	6.21589 **	0.0207
LRF ⇏ LOGWP	14.5460 ***	0.0001	11.2664 ***	0.0029	12.7836 ***	0.0017
LWP ⇏ LRF	5.86390 **	0.0104	2.30976	0.1428	1.31051	0.2646
LFER ⇏ LWP	14.0077 ***	0.0002	5.55914 **	0.0277	1.38081	0.2525
LWP ⇏ LFER	11.1690 ***	0.0006	1.33921	0.2596	14.7157 ***	0.0009
LPC ⇏ LWP	0.34197	0.7147	0.68055	0.4183	2.40316	0.1354
LWP ⇏ LPC	1.18261	0.3280	0.09899	0.7560	0.30692	0.5852
LWA ⇏ LWP	2.59154	0.1011	3.89070 *	0.0613	7.44369 **	0.0123
LOGWP ⇏ LWA	1.66343	0.2159	4.21314 *	0.0522	11.3607 ***	0.0028
LLF ⇏ LWP	1.83122	0.1874	0.00149	0.9695	4.15717 *	0.0537
LWP ⇏ LLF	0.25914	0.7744	2.30602	0.1431	0.15467	0.6979

Note: ⇏ indicates “does not cause Granger”, *** *p* value < 0.01, ** *p* value < 0.05, and * *p* value < 0.1.

## Data Availability

The data will be available on request.
